# Impact of Cold-Water Immersion Compared with Passive Recovery Following a Single Bout of Strenuous Exercise on Athletic Performance in Physically Active Participants: A Systematic Review with Meta-analysis and Meta-regression

**DOI:** 10.1007/s40279-022-01644-9

**Published:** 2022-02-14

**Authors:** Emma Moore, Joel T. Fuller, Jonathan D. Buckley, Siena Saunders, Shona L. Halson, James R. Broatch, Clint R. Bellenger

**Affiliations:** 1 Alliance for Research in Exercise, Nutrition and Activity (ARENA), University of South Australia, Adelaide, SA Australia; 2 Faculty of Medicine, Health and Human Sciences, Macquarie University, Sydney, NSW Australia; 3 School of Behavioural and Health Sciences, McAuley at Banyo, Brisbane, QLD Australia; 4 Institute for Health and Sport (IHES), Victoria University, Footscray, VIC Australia

## Abstract

**Background:**

Studies investigating the effects of cold-water immersion (CWI) on the recovery of athletic performance, perceptual measures and creatine kinase (CK) have reported mixed results in physically active populations.

**Objectives:**

The purpose of this systematic review was to investigate the effects of CWI on recovery of athletic performance, perceptual measures and CK following an acute bout of exercise in physically active populations.

**Study Design:**

Systematic review with meta-analysis and meta-regression.

**Methods:**

A systematic search was conducted in September 2021 using Medline, SPORTDiscus, Scopus, Web of Science, Cochrane Library, EmCare and Embase databases. Studies were included if they were peer reviewed and published in English, included participants who were involved in sport or deemed physically active, compared CWI with passive recovery methods following an acute bout of strenuous exercise and included athletic performance, athlete perception and CK outcome measures. Studies were divided into two strenuous exercise subgroups: eccentric exercise and high-intensity exercise. Random effects meta-analyses were used to determine standardised mean differences (SMD) with 95% confidence intervals. Meta-regression analyses were completed with water temperature and exposure durations as continuous moderator variables.

**Results:**

Fifty-two studies were included in the meta-analyses. CWI improved the recovery of muscular power 24 h after eccentric exercise (SMD 0.34 [95% CI 0.06–0.62]) and after high-intensity exercise (SMD 0.22 [95% CI 0.004–0.43]), and reduced serum CK (SMD − 0.85 [95% CI − 1.61 to − 0.08]) 24 h after high-intensity exercise. CWI also improved muscle soreness (SMD − 0.89 [95% CI − 1.48 to − 0.29]) and perceived feelings of recovery (SMD 0.66 [95% CI 0.29–1.03]) 24 h after high-intensity exercise. There was no significant influence on the recovery of strength performance following either eccentric or high-intensity exercise. Meta-regression indicated that shorter time and lower temperatures were related to the largest beneficial effects on serum CK (duration and temperature dose effects) and endurance performance (duration dose effects only) after high-intensity exercise.

**Conclusion:**

CWI was an effective recovery tool after high-intensity exercise, with positive outcomes occurring for muscular power, muscle soreness, CK, and perceived recovery 24 h after exercise. However, after eccentric exercise, CWI was only effective for positively influencing muscular power 24 h after exercise. Dose–response relationships emerged for positively influencing endurance performance and reducing serum CK, indicating that shorter durations and lower temperatures may improve the efficacy of CWI if used after high-intensity exercise.

**Funding:**

Emma Moore is supported by a Research Training Program (Domestic) Scholarship from the Australian Commonwealth Department of Education and Training.

**Protocol registration:**

Open Science Framework: 10.17605/OSF.IO/SRB9D.

**Supplementary Information:**

The online version contains supplementary material available at 10.1007/s40279-022-01644-9.

## Key Points

**Table:** 

Cold-water immersion is more likely to positively influence muscular power performance, but not muscular strength performance.
Cold-water immersion is more likely to positively influence muscular power performance, muscle soreness, serum creatine kinase, and perceived recovery after high-intensity exercise when compared with passive recovery.
Dose–response relationships indicate that *lower temperature* cold-water immersion may be more effective after high-intensity exercise for removal of serum creatine kinase.
Dose–response relationships indicate *shorter duration* cold-water immersion may be more effective after high-intensity exercise for endurance performance and removal of serum creatine kinase.

## Introduction

During acute (short-term) phases of training and competition, athletes are required to maximise their performance in competition while managing fatigue and soreness [1]. Therefore, recovery between exercise bouts is paramount to prepare for subsequent training sessions or competition events [2] and failure to adequately recover may lead to fatigue and reductions in performance [3]. Therefore, techniques have been developed to accelerate recovery and optimise performance, especially in scenarios where time for recovery may be limited, with the goal of assisting athletes to reach their peak performance in competition. Cold-water immersion (CWI) is a commonly performed recovery technique that can be cost effective and able to be utilised in many environments [4]. Studies have found that CWI is one of the most effective recovery strategies implemented by athletes, with an effectiveness rating of 4.3/5 [5].

The beneficial effects of CWI in recovery are thought to be largely mediated by increased hydrostatic pressure and/or a reduction in body/tissue temperature [6]. Resultant alterations in tissue blood flow, post-exercise fluid retention and metabolic activity may accelerate the recovery process via the reduction of muscle damage, swelling and inflammation, muscle spasm, pain, and thermal strain [4]. CWI may also improve mood due to endorphin release [7] and enhance perceptions of recovery by reducing muscular soreness via an analgesic effect [1].

Recently completed meta-analyses investigating the efficacy of CWI for promoting recovery from an acute bout of exercise have reported performance, perceptual and physiological benefits of CWI compared with passive recovery [8–11]. However, one review was limited in that it combined multiple timepoints of recovery (i.e. 24 h, 48 h, 72 h etc.) within a single analysis [10] such that the investigators could not identify a time course of recovery. These reviews also pooled data from multiple cooling methods (i.e. CWI, cooling packs, cryotherapy chambers) [10], and different exercise intervention modes (i.e. eccentric exercise and high-intensity exercise) [9–11], and both crossover and parallel studies with no consideration of the statistical differences between study designs. For example, many crossover studies do not report within-participant differences and provide only mean values for each treatment group, which reduces the precision of the results [12]. These variations resulted in high levels of heterogeneity within the analyses, and only one review attempted sub-group analyses to reduce heterogeneity, with limited effect [8]. While the time-based (24 h, 48 h, 72 h) and exercise intervention-based (eccentric and high-intensity exercise) subgroups showed some positive effects of CWI, in most of the analyses the heterogeneity was not reduced, and the low study numbers (< 3) in some subgroups minimised conclusions that could be made.

One review [9] attempted to elucidate whether there was a dose–response relationship between muscle soreness and CWI temperature or exposure duration in trained and untrained participants. However, that review arbitrarily clustered temperatures and durations into small sub-meta-analyses. This sub-meta-analyses approach combines results to obtain a summarised mean difference that only takes into account the methodological differences between studies (heterogeneity) [13, 14]. This review identified an ‘optimum’ CWI protocol of 11–15 ℃ for 11–15 min based on small study numbers (< 4 per sub-analysis), and this protocol has been subsequently used as the guideline for recovery prescription across various sporting populations despite the limited evidence. This review also only focused on the reduction of muscle soreness, ignoring other parameters of recovery. When making recommendations for appropriate protocols, the strength of evidence should incorporate more than just heterogeneity, but also include the number of participants and level of bias within the studies such as is seen in the Grading of Recommendations Assessment, Development and Evaluation (GRADE) method of grading evidence quality and strength of recommendations [15]. Therefore, the small study numbers (and subsequently participant numbers) weaken any definitive conclusions.

The aim of this review was to investigate the impact of CWI on recovery of physiological, perceptual, and athletic performance variables following an acute bout of strenuous exercise in physically active participants. Additionally, this review aimed to describe the recovery of these variables across multiple timepoints following exercise of differing modality, as well as identify dose–response effects of temperature and exposure duration using meta-regression. This review also aimed to provide systematic recommendations using GRADE criteria. Identifying protocols that aid recovery of specific athletic performance outcomes following strenuous exercise will allow appropriate prescription of CWI protocols for physically active individuals who wish to accelerate their recovery following exercise.

## Methods

### Design

This review followed the Preferred Reporting Items for Systematic Reviews and Meta-Analysis (PRISMA) statement for the reporting of systematic reviews and meta-analyses [16] and was prospectively registered with Open Science Framework (10.17605/OSF.IO/SRB9D).

### Search Strategy and Selection Criteria

Medline, SPORTDiscus, Scopus, Web of Science, Cochrane Library, EmCare and Embase databases were searched from inception until 20 September 2021 using the following search strategy, which was adapted for each database:

athlet* or sport* or exerci* or football* or soccer or hockey or basketball* or netball* or volleyball* or "track and field" or cycli* or running or runner* or swim* or handball or softball* or tennis or baseball or cross country or cricket or surf* or skiing or golf or hurdling or bicycling or boxing or gymnast* or martial arts or racquet sports or badminton or jogg* or walk* or weight lifting or lift* weights or weight?lift* or wrestling or resistance train* or endurance train* or interval train* or climb* or strength* train* or strength* program and (cold* or ice* or low* temp*) adj3 (bath* or hydrotherap* or immers* or submers* or submerg*)

Individual sport terms were included in the search strategy to ensure that studies that used sport simulations, training or games were captured in the search process; this ensured a vigorous search was completed.

Database search results were exported to Endnote^©^ (version 9.2, Thomson-Reuters, Toronto, CA, USA) and then uploaded to Covidence^©^ Systematic Review software (Veritas Health Innovations, Melbourne, VIC, Australia). All duplicates were removed before two reviewers independently screened titles and abstracts for eligibility. Full texts were obtained for the remaining articles and independently assessed for eligibility by two reviewers (EM, SS). Results from each reviewer were compared after each stage and any discrepancies were resolved by an independent reviewer. Reference lists of all eligible studies and any previous systematic reviews were checked to identify additional eligible studies that were not identified by the primary search (i.e. pearling).

Inclusion criteria were as follows: (i) peer-reviewed, randomised controlled trials published in the English language, (ii) participants were aged over 18 years and performed exercise regularly, (iii) protocols that used CWI within 15 min following a single bout of strenuous exercise (defined by the authors as an exercise bout that would cause muscle damage) with further immersions permitted to be completed on subsequent days, (iv) used passive recovery (no recovery intervention) as the comparator intervention (to determine the influence of CWI on recovery measures), (v) outcome measures included time to recovery of exercise performance (endurance, flexibility, muscular strength, muscular power [including jump performance, anaerobic power performance of < 10 s or sprint performance]) or physiological (creatine kinase [CK]) and perceptual markers of recovery (delayed onset muscle soreness [DOMS], perceived recovery) and (vi) outcome measures were performed at the following timepoints: 1 h after exercise, 24 h after exercise, 48 h after exercise, 72 h after exercise, 96 h after exercise and 168 h after exercise. Studies were excluded if they used combined treatments that may confound CWI results (e.g. combining CWI with compression garments, CWI with active recovery, CWI with nutritional supplements), training interventions involving more than one session of exercise, there were insufficient data to perform analysis or if data were presented in formats such as theses or conference abstracts.

### Risk of Bias

An assessment of methodological quality for the selected studies was undertaken using the randomised controlled trial (RCT) checklist from the Scottish Intercollegiate Guidelines Network (SIGN) [17]. The SIGN RCT checklist was developed to ensure a balance between methodological quality and practicality of use for authors and was used in the present review because it is specific to the design of included studies. Before commencing assessment, definitions provided by SIGN were clarified by the review team. Two reviewers (EM, SS) appraised each study based on these appraisal definitions, with any discrepancies resolved by an independent reviewer (JB). A grade of ‘yes’, ‘no’, ‘can’t say’ or ‘not applicable’ was issued for each appraisal item. ‘Yes’ and ‘not applicable’ answers were indicative of a lower risk of bias; therefore, the total frequency of ‘yes’ and ‘not applicable’ answers were tallied to indicate overall methodological quality. The quality of each study was labelled as ‘high quality’, ‘acceptable’, ‘low quality’ or ‘unacceptable’.

### Data Extraction

Data were extracted by one reviewer (EM) and entered in a standardised Microsoft Excel^©^ spreadsheet (V2105, Microsoft, Washington, USA). These data were independently crosschecked by another reviewer (SS) and any discrepancies were resolved through discussion. Further information was sought from study authors if all information could not be obtained from the full-text article. The extracted information included publication details (author information, publication date, country of origin), study methodology (sample size, exercise intervention, study type, assessment measures, comparison intervention), participant information (age, sex, height, body mass, sport, training history), CWI protocol (temperature, duration, number of immersions, depth of immersion, body position during immersion, timing of immersion post-exercise), passive recovery protocol (temperature of environment, duration, body position during protocol), and assessment measures (test, units, measurements at various timepoints, effect sizes, confidence intervals, *p*-values).

### Statistical Considerations

Summary effect sizes were presented for each study using standardised mean difference (SMD) for comparing the effect between CWI and passive recovery. SMDs were calculated using Hedges’ h correction for positive bias. The precision of the effect sizes was described using 95% confidence intervals (CI) whenever sufficient information was provided by the study authors. For the purpose of this review, effect sizes were presented for each study and were considered trivial (SMD < 0.2), small (SMD 0.20–0.60), moderate (SMD 0.61–1.20), large (SMD 1.21–2.00) and very large (SMD > 2.01) [18].

Random-effects meta-analyses and meta-regression were performed using restricted maximum likelihood estimation with the Metafor statistical package in R software (version 3.4.3, R Foundation for Statistical Computing). Weighting of study effects was based on the inverse variance method. Separate analyses were performed for exercise mode subgroups (high-intensity exercise vs resistance-based eccentric muscle contraction) to identify whether the effects of differing exercise modalities alter the recovery timeline. Both CWI duration and temperature were considered as continuous moderator variables due to the various combinations of duration and temperature evident in the data synthesis. Study ID was included as a random factor to account for studies that reported multiple CWI versus passive recovery comparisons (i.e. different CWI temperatures).

The methods described by Elbourne et al. [12] were used to combine data from parallel and crossover studies in the meta-analyses whenever possible. This was possible for crossover studies that reported treatment effect with an accompanying confidence interval, standard error, or *p*-value from an appropriate statistical analysis of paired data. Crossover studies that did not provide this information (i.e. they only reported mean and standard deviation for each treatment condition) were also included if it was possible to estimate the standard error of the paired difference using information available from other included crossover studies of the same outcome measure. This estimation process is described in detailed by Elbourne et al. [12]. In brief, the correlation between treatment and comparison conditions can be estimated from the included crossover studies that provide complete information from paired analysis. This estimated correlation value can then be used to impute a standard error for the treatment effect from crossover studies that do not provide information about treatment effect variance. The lowest available correlation was used for this imputation when multiple correlations were available for a review outcome.

Muscular power outcomes for jump, sprint and anaerobic power of < 10 s were pooled for the purpose of analysis to increase study numbers. Despite potential differences in the kinetics of recovery, it was considered appropriate to combine these outcomes to evaluate the effects on recovery of the phosphagen energy system.

Statistical heterogeneity within each meta-analysis was investigated using *I*^2^ statistics, which indicated the consistency of the study effects between the included studies [19]. Statistical heterogeneity (i.e. inconsistency) was considered low (*I*^2^ < 25%), moderate (*I*^2^ 25–49%) and high (*I*^2^ > 50%) [19]. The overall quality of the evidence synthesis was rated high, moderate, low or very low using the GRADE system [15]. The quality rating was downgraded one level from high for each of the following limitations: total number of unique participants was < 100 (imprecision), high statistical heterogeneity (inconsistency) and more than 50% of the studies in the meta-analysis deemed to be low quality.

## Results

### Search Results

**Fig. 1:**
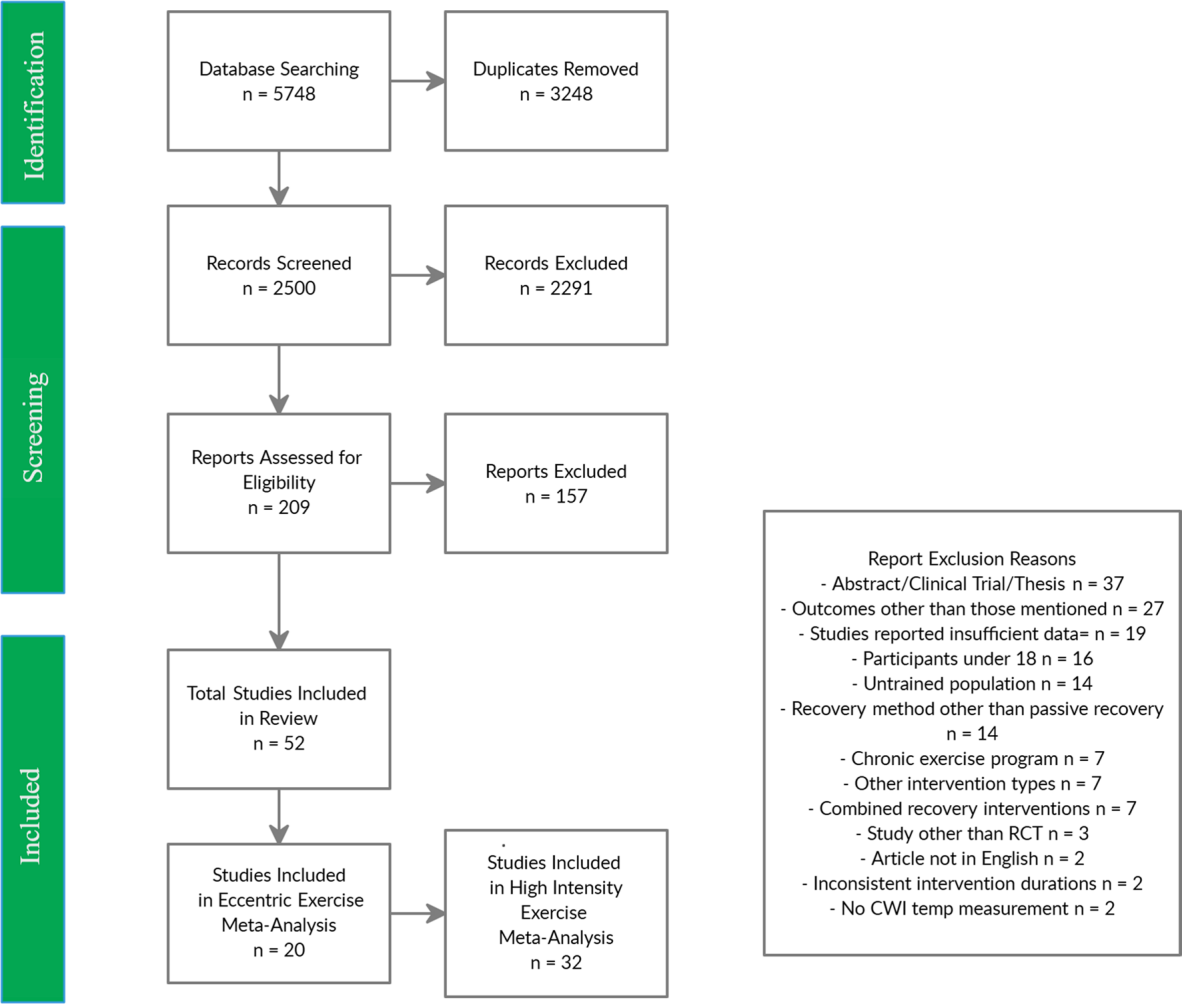
PRISMA flowchart for screening of articles. *CWI* cold-water immersion, *RCT* randomised controlled trial

Systematic database searches identified 4849 potential studies and pearling of previous reviews identified six more potential studies for inclusion. Following the removal of duplicates and ineligible articles, 52 studies were included in this review. Several studies met the inclusion criteria but were excluded due to missing/unusable data (where authors were not able to be contacted) or outcomes measured at timepoints not included in the meta-analysis [20–38]. A complete overview of the screening process can be found in Fig. 1. A complete overview of articles identified for inclusion in the review can be found in Table 1.

**Table 1:** Overview of included studies

Study	Study type	Subject N, sex	Exercise protocol	CWI group	Outcome measures	Timing of measures
*Eccentric exercise interventions*						
Adamczyk et al. 2016 [39]	Parallel	36 males	1-min jumping from squat position	8 ℃ 3 min	DOMS	24 h, 48 h, 72 h, 96 h
Amir et al. 2016 [40]	Parallel	16 males	10 × 10 CMJ	15 ℃ 15 min	CK; DOMS; flexibility (knee ROM); strength (peak concentric force)	24 h, 48 h, 72 h, 96 h
Argus et al. 2017 [41]	Crossover	13 males	Resistance training protocol (3 × 5 deadlifts; 3 × 10 back squats; 3 × 10 bench press; 3 × 10 BB lunge; 3 × 10 BB bent over row)	15 ℃ 14 min	DOMS; power (CMJ); strength (peak isometric force)	1 h
Doeringer et al. 2018 [42]	Parallel	22 males & females	Plyometric training protocol (change of direction, tuck jumps, zig zag hops, depth jumps, depth presses)	10 ℃ 25 min	DOMS; flexibility (sit and reach); power (CMJ)	24 h, 48 h
Eston et al. 1999 [43]	Parallel	15 females	8 × 5 eccentric maximal voluntary contractions	15 ℃ 15 min	CK; strength (peak isometric force)	24 h, 48 h, 72 h
Goodall and Howatson 2008 [44]	Parallel	18 males	5 × 20 drop jumps	15 ℃ 12 min	CK; DOMS; strength (peak isometric force)	24 h, 48 h, 72 h, 96 h
Hassan 2011 [45]	Parallel	60 males	10 × 10 eccentric hamstring contractions	20 ℃ 30 min	CK	1 h
Hohenauer et al. 2019 [46]	Parallel	28 females	5 × 20 drop jumps	10 ℃ 10 min	DOMS; power (CMJ); strength (peak isometric force)	1 h, 24 h, 48 h, 72 h
Howatson et al. 2009 [47]	Parallel	16 males	5 × 20 drop jumps	15 ℃ 12 min	CK; DOMS; flexibility (knee ROM); strength (peak isometric force)	24 h, 48 h, 72 h, 96 h
Jajtner et al. 2015 [48]	Parallel	30 males	Lower-body resistance programme (4 sets of squats, deadlifts, BB split squats [at 70–80% of 1RM])	10–12 ℃ 10 min	CK	24 h, 48 h
Jakeman et al. 2009 [49]	Parallel	18 females	10 × 10 countermovement jumps	10 ℃ 10 min	CK; DOMS; strength (peak concentric force)	1 h, 24 h, 48 h, 72 h, 96 h
Machado et al. 2017 [50]	Parallel	60 males	5 × 15 eccentric knee contractions	9 ℃ 15 min 14 ℃ 15 min	CK; DOMS; PR; strength (peak isometric force)	24 h, 48 h, 72 h, 96 h
Missau et al. 2018 [51]	Parallel	13 males	Resistance training protocol (4 × 10RM extensor chair, squats, leg press)	15 ℃ 10 min	CK; DOMS	24 h
Paddon-Jones and Quigley 1997 [52]	Crossover	8 males	8 × 8 eccentric dumbbell curls	5 ℃ 20 min	Strength (peak isometric force)	24 h, 48 h, 72 h, 96 h
Rose et al. 2014 [53]	Crossover	13 males	200 maximal leg extension contractions	11 ℃ 3 min	DOMS; strength (peak isometric force)	24 h, 48 h, 168 h
Sánchez-Ureña et al. 2018 [54]	Parallel	39 males	8 × 30-s countermovement jump repetitions	12 ℃ 12 min 12 ℃ 6 × 2 min	Power (CMJ)	24 h, 48 h
Siqueira et al. 2018 [55]	Parallel	29 males	5 × 20 drop jumps	10 ℃ 20 min	CK; DOMS; power (CMJ); strength (peak isometric force)	24 h, 48 h, 72 h, 96 h, 168 h
Vaile et al. 2008 [56]	Parallel	38 males	5 × 10 leg press at 120% 1RM, 2 × 10 leg press at 100% 1RM	15 ℃ 14 min	CK; DOMS; power (squat jump); strength (peak isometric force)	24 h, 48 h, 72 h
Vanderlei et al. 2017 [57]	Parallel	105 males	10 × 10 jumps followed by Wingate anaerobic bike protocol (5-min warmup, 30-s test [0.075 Kp/kg body weight])	9 ℃ 5 min 14 ℃ 5 min 9 ℃ 15 min 14 ℃ 15 min	CK; DOMS; PR	24 h, 48 h, 72 h, 96 h
Vieira et al. 2016 [58]	Parallel	42 males	5 × 20 drop jumps	5 ℃ 20 min 15 ℃ 20 min	CK; DOMS; power (CMJ); strength (peak isometric force)	24 h, 48 h, 72 h, 96 h, 168 h
*High-intensity exercise interventions*						
Anderson et al. 2018 [59]	Crossover	9 males	Intermittent running protocol (2 × 21 min treadmill—alternating 1 min 6 km/h, 1 min 15 km/h, 1 min 18 km/h)	14 ℃ 12 min 5 ℃ 12 min	CK; DOMS; power (peak 10-s cycling at 10% body mass)	24 h, 48 h, 72 h
Bailey et al. 2007 [60]	Parallel	20 males	Longborough intermittent shuttle test (5 sets of 15 min of varying intensity [walk, jog, run])	10 ℃ 10 min	CK; DOMS; strength (peak isometric force)	1 h, 24 h, 48 h, 168 h
Barber et al. 2020 [61]	Parallel	16 males	Rugby Union match simulation	10 ℃ 2 × 5 min	CK; DOMS; power (CMJ); strength (peak isometric force)	1 h, 24 h, 48 h
Bosak et al. 2009 [62]	Crossover	12 males and females	5-km run	15.5 ℃ 12 min	Endurance (5-km run)	24 h
Brophy-Williams et al. 2011 [63]	Crossover	8 males	High-intensity interval session (8 × 3-min treadmill running at 90% VO_2_max)	15 ℃ 15 min	DOMS; PR; endurance (yo-yo shuttle run)	24 h
Crowther et al. 2017 [64]	Crossover	34 males	3 × 15-min simulated team sport circuit (walk, jog, stride, run, sprint, agility, tackling, bumping)	15 ℃ 14 min	DOMS; PR; flexibility (sit and reach test); power (CMJ, 20-m sprint)	1 h, 24 h, 48 h
Crystal et al. 2013 [65]	Parallel	20 males	40 min downhill running	5 ℃ 20 min	DOMS; strength (peak isometric force)	1 h, 24 h, 48 h, 72 h
Dantas et al. 2020 [66]	Parallel	30 males	10-km run	10 ℃ 10 min	CK; DOMS; power (triple hop jump); strength (peak concentric force)	24 h
Delextrat et al. 2013 [67]	Crossover	16 males & females	Basketball match	11 ℃ 5 × 2 min	Power (CMJ, RSA over 30 m)	24 h
Elias et al. 2012 [68]	Crossover	14 males	Australian football training session	12 ℃ 14 min	DOMS; power (CMJ, RSA over 20 m)	1 h, 24 h, 48 h
Elias et al. 2013 [69]	Parallel	24 males	Australian football match	12 ℃ 14 min	DOMS; power (CMJ, RSA over 20 m)	1 h, 24 h, 48 h
Fonseca et al. 2016 [70]	Crossover	8 males	Jiu-Jitsu training	6 ℃ 4 × 4 min	CK; DOMS; PR; power (CMJ)	24 h, 48 h
Getto and Golden 2013 [71]	Parallel	23 males & females	Team sport conditioning session (sprinting, plyometric bounding and hopping)	10 ℃ 10 min	DOMS; power (CMJ, 20-m sprint)	24 h
Higgins et al. 2013 (1) [72]	Parallel	24 males	Simulated rugby union game	10–12 ℃ 2 × 5 min	Flexibility (sit and reach); power (CMJ)	1 h, 24 h, 48 h
Higgins et al. 2013 (2) [73]	Parallel	24 males	Simulated rugby union game	10–12 ℃ 2 × 5 min	DOMS; flexibility (sit and reach); power (CMJ)	1 h, 48 h, 72 h, 96 h
Ingram et al. 2009 [74]	Crossover	11 males	Simulated team sport exercise (4 × 20-min intermittent running; beep test shuttle runs until failure)	10 ℃ 2 × 5 min	CK; DOMS; power (20-m sprint); strength (peak isometric force)	24 h, 48 h
Jones et al. 2013 [75]	Crossover	10 males	Rugby Sevens simulation	10 ℃ 10 min	PR; power (CMJ)	24 h
Lane and Wenger 2004 [76]	Crossover	10 males	18-min intermittent cycling (22 repetitions of varying durations with a 1:5 work-rest ratio)	15 ℃ 15 min	Endurance (18-min intermittent cycling)	24 h
Leeder et al. 2015 [77]	Parallel	24 males	Longborough intermittent shuttle test (5 sets of 15 min of varying intensity [walk, jog, run])	14 ℃ 14 min	CK; DOMS; power (CMJ); strength (peak isometric force)	24 h, 48 h 72 h
Leeder et al. 2019 [78]	Parallel	21 males	Longborough intermittent shuttle test (5 sets of 15 min of varying intensity [walk, jog, run])	14 ℃ 14 min	CK; power (CMJ); strength (peak isometric force)	48 h, 96 h
Lindsay et al. 2017 [79]	Parallel	15 males	90-min MMA contest preparation training	10 ℃ 15 min	DOMS; power (CMJ)	1 h, 24 h
Minett et al. 2014 [80]	Crossover	9 males	Intermittent sprint bouts (2 × 35 min—5 sets of 6 × 15-m sprints, 5-min periods of 15-m shuttle runs)	10 ℃ 20 min	CK; DOMS; strength (peak isometric force)	1 h, 24 h
Moriera et al. 2015 [81]	Crossover	10 males	Futsal game	15 ℃ 12 min	DOMS; power (CMJ, RSA over 30 m)	24 h
Pointon and Duffield 2012 [82]	Crossover	10 males	Intermittent running protocol with tackling (2 × 30-min bouts with 15-m and 10-m repeated sprints)	9 ℃ 2 × 9 min	DOMS; strength (peak isometric force)	24 h
Pournot et al. 2011 [83]	Parallel	41 males	Intermittent exercise protocol (2 × 10-min bouts of alternating 30 × CMJ and 30-s rowing)	10 ℃ 15 min	CK; DOMS; power (CMJ); strength (peak isometric force)	1 h, 24 h
Rupp et al. 2012 [84]	Parallel	22 males & females	Yo-Yo intermittent recovery test	12 ℃ 15 min	Endurance (yo-yo intermittent recovery test); power (CMJ)	24 h, 48 h
Stenson et al. 2017 [85]	Crossover	9 males	Interval running protocol (8 × 1200-m runs at 75% VO_2_peak)	12 ℃ 12 min	DOMS; flexibility (knee ROM); endurance (5-km time trial)	24 h
Tabben et al. 2018 [86]	Crossover	12 males	Simulated MMA competition	10 ℃ 15 min	CK; power (CMJ, 10-m sprint)	24 h
Takeda et al. 2014 [87]	Crossover	20 males	Rugby match simulation	15 ℃ 10 min	CK; power (CMJ, 50-m sprint)	24 h
Vaile et al. 2008 [88]	Crossover	12 males	Cycling HIIT (9 sets of sprint cycling of varying durations with varying work/rest ratios)	15 ℃ 14 min	Endurance (9-min time trial)	24 h, 48 h, 72 h, 96 h
White et al. 2014 [89]	Crossover	8 males	HIIT sprint protocol (12 maximal sprints of 120 m every 3 min)	20 ℃ 10 min 20 ℃ 30 min 10 ℃ 10 min 10 ℃ 30 min	DOMS	24 h, 48 h
Wiewelhove et al. 2018 [90]	Parallel	46 males	Half marathon	15 ℃ 15 min	CK; DOMS; PR; power (CMJ)	24 h

*℃* degrees Celsius, *BB* barbell, *CK* creatine kinase, *CMJ* counter movement jump, *DOMS* delayed onset muscle soreness, *h* hours, *HIIT* high-intensity interval training, *km* kilometres, *m* metres, *min* minutes, *MMA* mixed martial arts, *PR* perceived recovery, *RM* repetition-max, *ROM* range of motion, *RSA* repeat sprint ability

### Risk of Bias

Based upon the agreed criteria of the SIGN RCT checklist, reviewers deemed 11 (21%) studies to be high quality [42, 50, 51, 54, 57, 58, 60, 66, 77, 79, 89], 37 (69%) to be acceptable quality [39–41, 43–49, 52, 53, 55, 56, 59, 61, 64, 65, 67–76, 78, 80–84, 86–88] and four (10%) studies to be low quality [62, 63, 85, 90]. The noteworthy results from the risk of bias analysis indicated that concealment of the treatment groups from the research group was rarely completed; only four studies concealed treatment groups from the research team [50, 57, 66, 89]. Reporting of randomisation protocols of treatment groups was also poor for most studies; only 11 studies adequately reported randomisation protocols [42, 50, 51, 54, 57, 58, 60, 66, 77, 79, 89]. Results of the risk of bias analysis for each individual study can be found in Online Supplement 1 of the electronic supplementary material (ESM). No study reported any conflict of interest.

### Meta-analysis

#### The Effects of Cold-Water Immersion (CWI) on Recovery of Power Performance

**Fig. 2:**
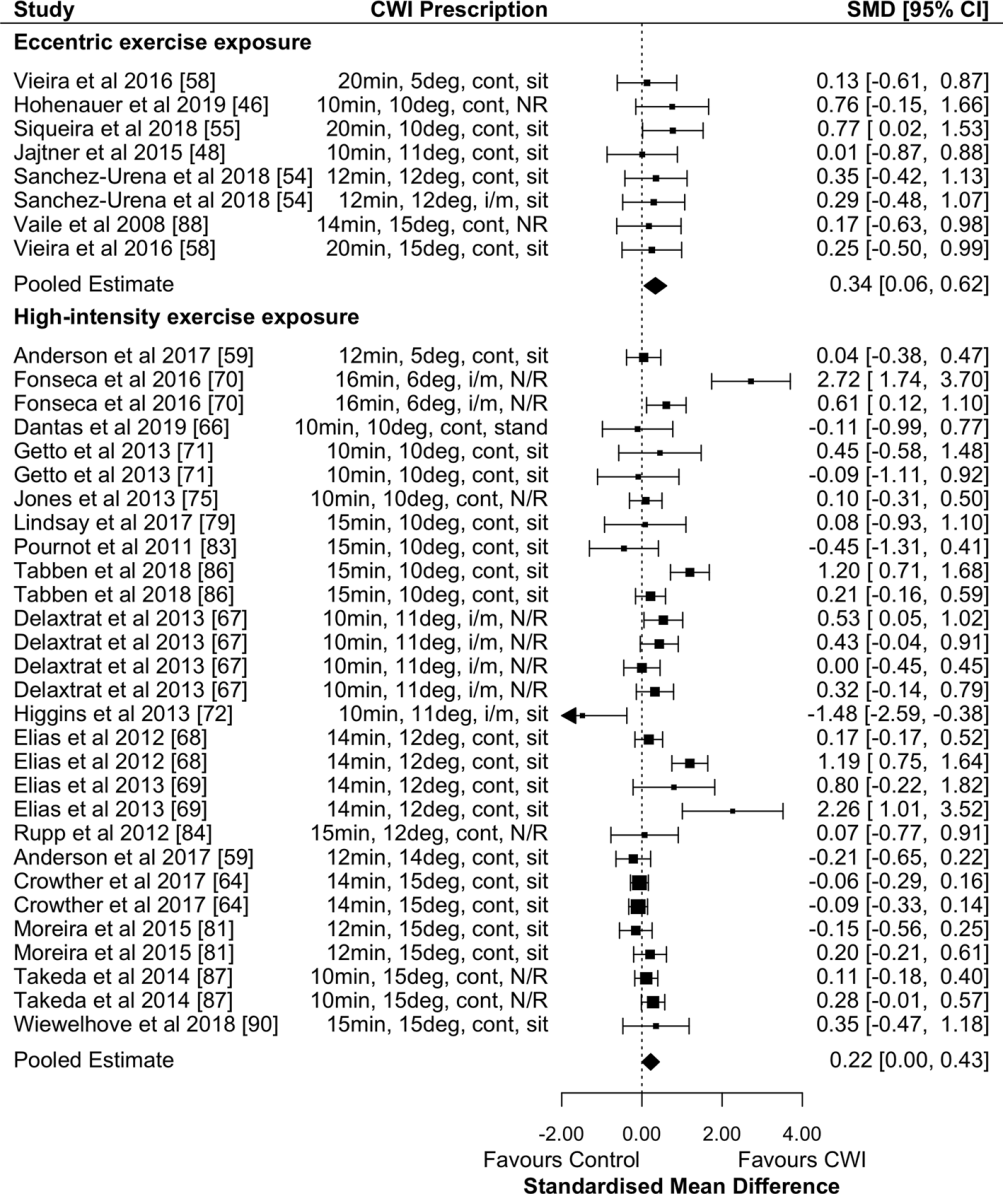
Forest plot illustrating the influence of CWI compared with passive recovery 24 h after exercise on muscular power performance (stratified by exercise intervention modality).*CI* confidence interval, *CWI* cold-water immersion, *SMD* standardised mean difference

CWI was effective in promoting the recovery of muscular power 24 h after eccentric exercise (Fig. 2; Table 2; small effects; *p* = 0.018; GRADE = high). There were significant small effects at 48 h, moderate effects at 72 h (Table 2; both timepoints GRADE = high) and non-significant small effects at 96 and 168 h (Table 2; both timepoints GRADE = moderate) in favour of CWI. Water temperature and exposure duration had no significant moderating effects at any timepoint.

**Table 2:** Meta-analysis summary

Outcome	Summary of findings					Quality of evidence synthesis (GRADE)			
	*K*(*k*)	*N*(*n*)	SMD (95% CI)	*p*-value	*I*^2^ (%)	Imprecision	Inconsistency	Risk of bias	Overall quality
CK 1 h (ecc)	2 (2)	58 (58)	0.85 (− 0.93 to 2.63)	0.349	87.5	− 1	− 1	None	Low
CK 1 h (hit)	Data could not be pooled (*K*(*k*) = 1(1))								
CK 24 h (ecc)	11 (16)	373 (470)	0.12 (− 0.31 to 0.54)	0.595	63.1	None	− 1	None	Moderate
CK 24 h (hit)	5 (6)	105 (113)	− 0.85 (− 1.61 to − 0.08)	0.030	58.6	None	− 1	None	Moderate
CK 48 h (ecc)	11 (16)	373 (470)	− 0.04 (− 0.37 to 0.30)	0.834	50.6	None	− 1	None	Moderate
CK 48 h (hit)	4 (5)	81 (89)	− 1.36 (− 2.51 to − 0.20)	0.022	77.3	− 1	− 1	None	Low
CK 72 h (ecc)	10 (15)	353 (450)	− 0.15 (− 0.57 to 0.27)	0.494	61.5	None	− 1	None	Moderate
CK 72 h (hit)	Data could not be pooled (*K*(*k*) = 1(2))								
CK 96 h (ecc)	6 (10)	243 (326)	− 0.04 (− 0.43 to 0.35)	0.839	39.4	None	None	None	High
CK 96 h (hit)	Data could not be pooled (*K*(*k*) = 1(1))								
CK 168 h (ecc)	2 (3)	71 (85)	− 0.62 (− 1.05 to − 0.18)	0.006	0	− 1	None	None	Moderate
CK 168 h (hit)	No studies								
DOMS 1 h (ecc)	4 (4)	64 (90)	0.00 (− 1.09 to 1.10)	0.998	69.4	− 1	− 1	None	Moderate
DOMS 1 h (hit)	9 (10)	149 (229)	− 1.10 (− 1.81 to − 0.40)	0.002	78.1	None	− 1	None	Moderate
DOMS 24 h (ecc)	13 (17)	401 (497)	− 0.51 (− 1.10 to 0.09)	0.094	75.9	None	− 1	None	Moderate
DOMS 24 h (hit)	20 (25)	297 (497)	− 0.89 (− 1.48 to − 0.29)	0.003	77.4	None	− 1	None	Moderate
DOMS 48 h (ecc)	12 (16)	375 (471)	− 0.48 (− 0.79 to − 0.16)	0.003	49.1	None	None	None	High
DOMS 48 h (hit)	11 (16)	164 (330)	− 0.82 (− 1.57 to − 0.07)	0.031	83.2	None	− 1	None	Moderate
DOMS 72 h (ecc)	10 (14)	340 (423)	− 0.55 (− 0.86 to − 0.23)	0.001	33.4	None	None	None	High
DOMS 72 h (hit)	4 (6)	69 (104)	0.09 (− 0.40 to 0.59)	0.718	23.9	− 1	None	None	Moderate
DOMS 96 h (ecc)	8 (12)	296 (379)	− 0.41 (− 0.62 to − 0.21)	< 0.001	0	None	None	None	High
DOMS 96 h (hit)	Data could not be pooled (*K*(*k*) = 1(1))								
DOMS 168 h (ecc)	2 (2)	42 (55)	− 0.78 (− 1.87 to 0.31)	0.160	75.0	− 1	− 1	None	Low
DOMS 168 h (hit)	Data could not be pooled (*K*(*k*) = 1(1))								
Power 1 h (ecc)	2 (2)	33 (46)	− 0.25 (− 0.58 to 0.07)	0.123	9.9	− 1	None	None	Moderate
Power 1 h (hit)	4 (5)	84 (174)	− 0.22 (− 0.52 to 0.09)	0.160	14.3	− 1	None	None	Moderate
Power 24 h (ecc)	6 (8)	174 (201)	0.34 (0.06 to 0.62)	0.018	0	None	None	None	High
Power 24 h (hit)	17 (29)	278 (676)	0.22 (0.004 to 0.43)	0.046	74.6	None	− 1	None	Moderate
Power 48 h (ecc)	7 (10)	196 (245)	0.47 (0.22 to 0.73)	< 0.001	0	None	None	None	High
Power 48 h (hit)	10 (14)	163 (357)	− 0.02 (− 0.12 to 0.07)	0.608	4.8	None	None	None	High
Power 72 h (ecc)	4 (5)	115 (129)	0.62 (0.27 to 0.98)	0.001	0	None	None	None	High
Power 72 h (hit)	2 (3)	25 (52)	− 0.01 (− 0.51 to 0.49)	0.969	0	− 1	None	None	Moderate
Power 96 h (ecc)	2 (3)	71 (85)	0.41 (− 0.02 to 0.84)	0.062	0	− 1	None	None	Moderate
Power 96 h (hit)	2 (2)	37 (37)	0.05 (− 0.59 to 0.70)	0.873	0	− 1	None	None	Moderate
Power 168 h (ecc)	2 (3)	71 (85)	0.36 (− 0.23 to 0.95)	0.228	0	− 1	None	None	Moderate
Power 168 h (hit)	Data could not be pooled (*K*(*k*) = 1(1))								
Strength 1 h (ecc)	2 (2)	38 (38)	2.31 (− 4.25 to 8.88)	0.490	97.2	− 1	− 1	None	Low
Strength 1 h (hit)	2 (2)	42 (42)	− 0.11 (− 0.72 to 0.50)	0.720	0	− 1	None	None	Moderate
Strength 24 h (ecc)	8 (10)	224 (258)	0.74 (− 0.78 to 2.26)	0.340	76.0	None	− 1	None	Moderate
Strength 24 h (hit)	3 (3)	62 (62)	0.12 (− 0.38 to 0.62)	0.637	0	− 1	None	None	Moderate
Strength 48 h (ecc)	8 (10)	224 (258)	0.84 (− 0.89 to 2.57)	0.339	78.9	None	− 1	None	Moderate
Strength 48 h (hit)	Data could not be pooled (*K*(*k*) = 1(1))								
Strength 72 h (ecc)	8 (10)	224 (258)	0.64 (− 0.51 to 1.79)	0.276	74.9	None	− 1	None	Moderate
Strength 72 h (hit)	Data could not be pooled (*K*(*k*) = 1(1))								
Strength 96 h (ecc)	5 (7)	165 (199)	− 0.30 (− 0.78 to 0.17)	0.212	36.8	None	None	None	High
Strength 96 h (hit)	No studies								
Strength 168 h (ecc)	2 (3)	71 (85)	− 0.09 (− 0.51 to 0.34)	0.691	0	− 1	None	None	Moderate
Strength 168 h (hit)	No studies								
Perceived recovery 24 h (ecc)	2 (6)	159 (242)	0.15 (− 0.29 to 0.59)	0.493	0	None	None	None	High
Perceived recovery 24 h (hit)	5 (5)	79 (135)	0.66 (0.29 to 1.03)	0.001	34.3	− 1	None	None	Moderate
Perceived recovery 48 h (ecc)	2 (6)	159 (242)	0.14 (− 0.11 to 0.39)	0.274	0	None	None	None	High
Perceived recovery 48 h (hit)	2 (2)	38 (76)	− 0.02 (− 0.38 to 0.34)	0.901	0	− 1	None	None	High
Perceived recovery 72 h (ecc)	2 (6)	159 (242)	0.55 (0.29 to 0.80)	< 0.001	0	None	None	None	High
Perceived recovery 72 h (hit)	No studies								
Perceived recovery 96 h (ecc)	2 (6)	159 (242)	0.38 (0.12 to 0.64)	0.004	2.9	None	None	None	High
Perceived recovery 96 h (hit)	No studies								
Endurance 24 h (ecc)	No studies								
Endurance 24 h (hit)	5 (5)	51 (102)	− 0.14 (− 0.40 to 0.13)	0.316	73.6	− 1	− 1	− 1	Very low
Endurance 48 h (ecc)	No studies								
Endurance 48 h (hit)	2 (2)	24 (46)	0.12 (− 0.16 to 0.40)	0.400	0	− 1	None	None	Moderate
Flexibility 1 h (ecc)	No studies								
Flexibility 1 h (hit)	3 (3)	62 (92)	2.14 (− 1.77 to 6.06)	0.283	92.9	− 1	− 1	None	Low
Flexibility 24 h (ecc)	Data could not be pooled (*K*(*k*) = 1(1))								
Flexibility 24 h (hit)	3 (3)	55 (94)	1.52 (− 1.78 to 4.82)	0.367	92.6	− 1	− 1	None	Low
Flexibility 48 h (ecc)	2 (2)	38 (38)	0.23 (− 0.41 to 0.87)	0.481	0	− 1	None	None	Moderate
Flexibility 48 h (hit)	3 (3)	62 (92)	2.23 (− 1.86 to 6.32)	0.286	93.3	− 1	− 1	None	Low
Flexibility 72 h (ecc)	Data could not be pooled (*K*(*k*) = 1(1))								
Flexibility 72 h (hit)	Data could not be pooled (*K*(*k*) = 1(1))								
Flexibility 96 h (ecc)	Data could not be pooled (*K*(*k*) = 1(1))								
Flexibility 96 h (hit)	Data could not be pooled (*K*(*k*) = 1(1))								

*CK* creatine kinase, *DOMS* delayed onset muscle soreness, *ecc* eccentric exercise, *GRADE* Grading of Recommendations Assessment, Development and Evaluation, *h* hour, *hit* high-intensity exercise, *K(k)* unique studies (observation points), *N(n)* unique participants (observation points), *SMD* standardised mean difference, *95% CI* 95% confidence interval

CWI was effective in promoting the recovery of muscular power 24 h after high-intensity exercise (Fig. 2; Table 2; small effects; *p* = 0.046; GRADE = moderate). Other timepoints found non-significant small effects in favour of passive recovery at 1 h (Table 2; GRADE = moderate) and no effect at 48 h (Table 2; GRADE = high), 72 h and 96 h (Table 2; both timepoints GRADE = moderate). Water temperature and exposure duration had no significant moderating effects at any timepoint.

#### The Effects of CWI on Recovery of Strength Performance

**Fig. 3:**
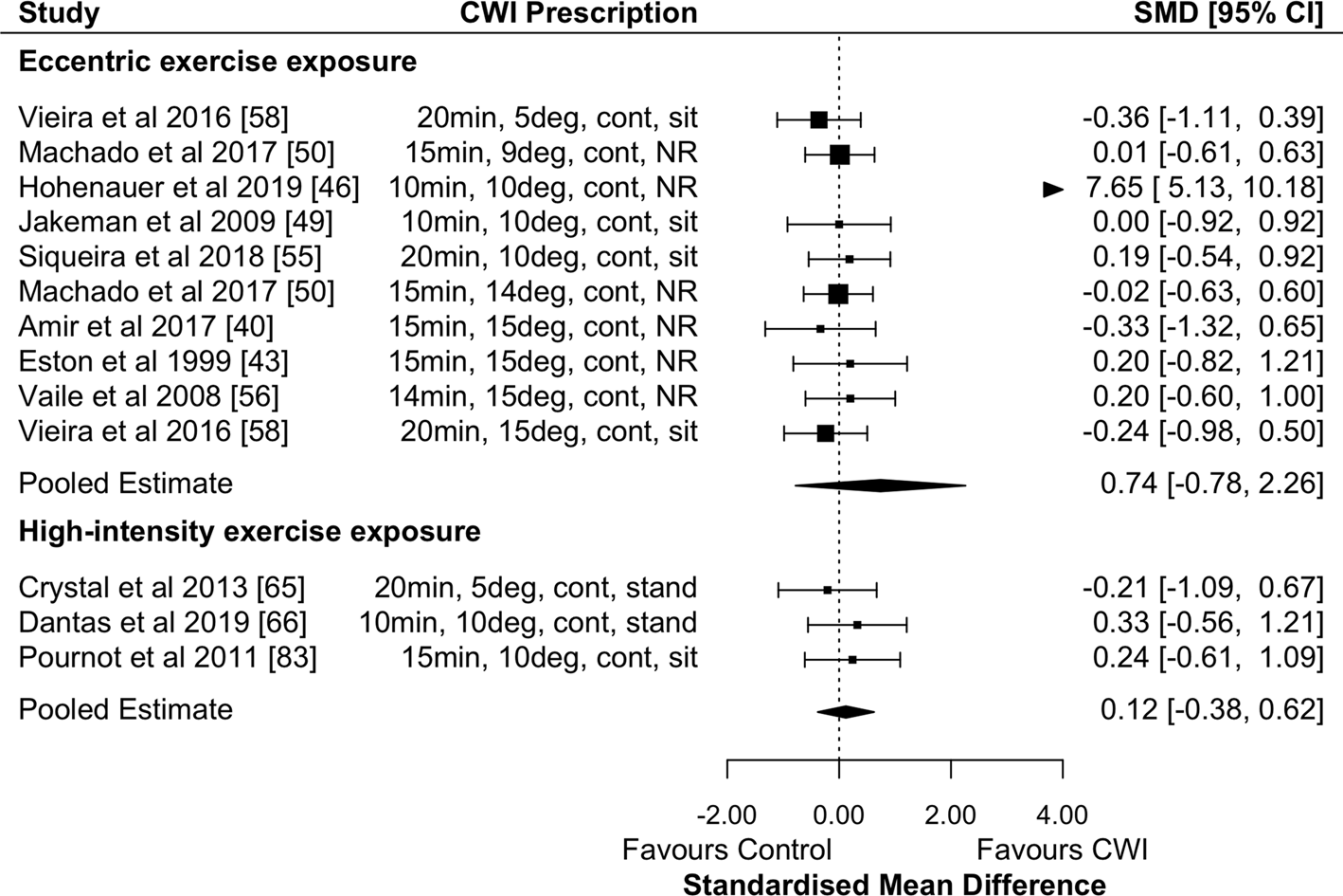
Forest plot illustrating the influence of CWI compared with passive recovery 24 h after exercise on strength performance (stratified by exercise intervention modality). *CI* confidence interval, *CWI* cold-water immersion, *SMD* standardised mean difference

CWI was not effective in promoting the recovery of strength performance 24 h after eccentric exercise (Fig. 3; Table 2; moderate effect; *p* = 0.34; GRADE = moderate). Other timepoints found non-significant very large effects at 1 h (Table 2; GRADE = low) and moderate effects at 48 and 72 h (Table 2; both timepoints GRADE = moderate). There were no significant moderating effects of water temperature or exposure duration at any timepoint.

There was no effect of CWI on the recovery of strength performance 24 h after high-intensity exercise (Fig. 3; Table 2; trivial effect; *p* = 0.64; GRADE = moderate) and water temperature and exposure duration did not have significant moderating effects.

#### The Effects of CWI on Recovery of Endurance Exercise Performance

There were no eccentric exercise studies that analysed the effects of CWI on endurance exercise performance.

**Fig. 4:**
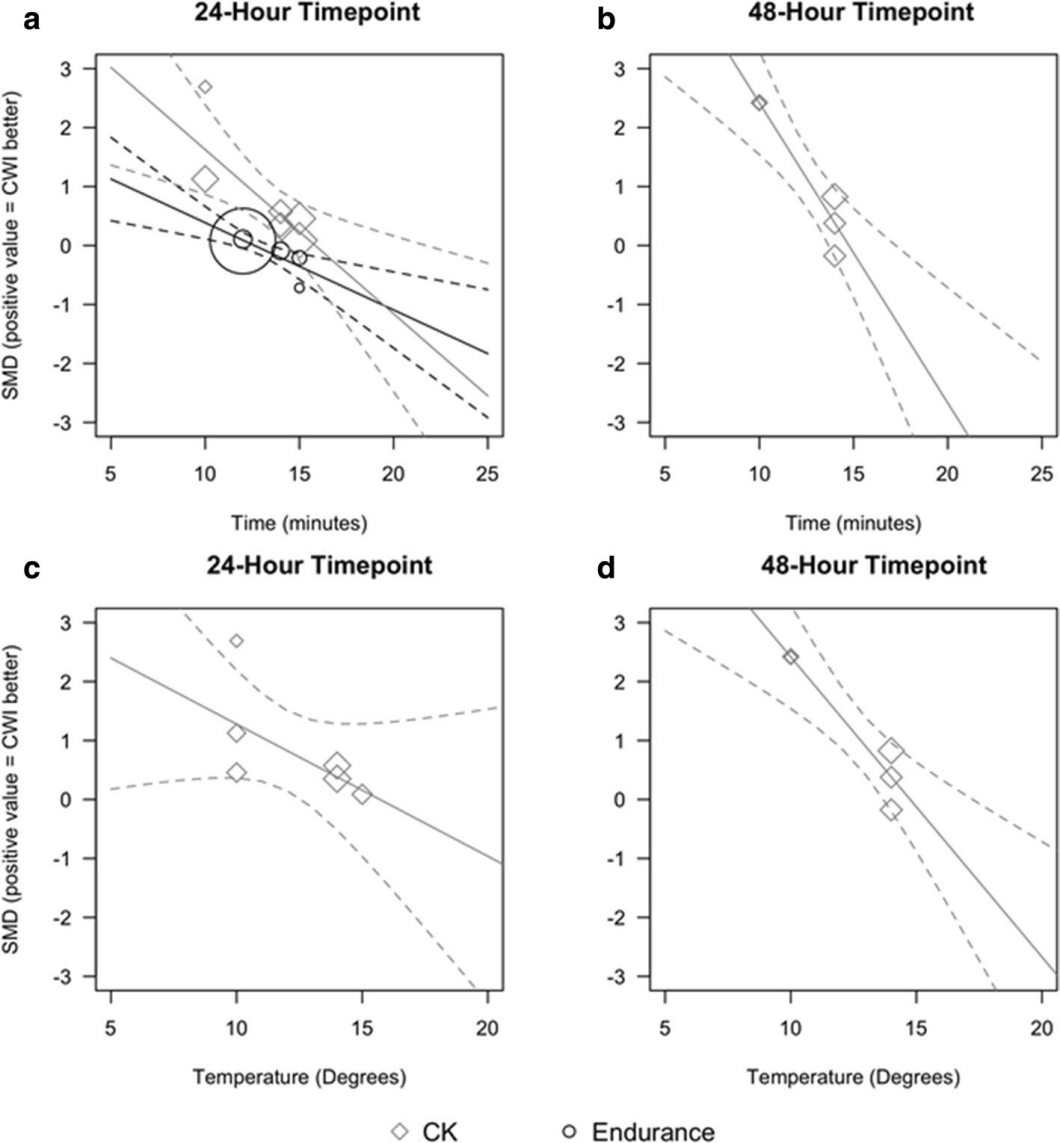
Meta-regression plots illustrating the influence of CWI duration and temperature on endurance performance and removal of serum CK from the blood at 24 h and 48 h after exercise. **a** Effect of time at 24 h after exercise; **b** effect of time at 48 h after exercise; **c** effect of temperature at 24 h after exercise; **d** effect of temperature at 48 h after exercise. *CK* creatine kinase, *CWI* cold-water immersion, *SMD* standardised mean difference

CWI was not effective in promoting recovery of endurance performance following high-intensity exercise. At 24 and 48 h, there were non-significant trivial effects (Table 2; GRADE 24 h = very low; GRADE 48 h = moderate). There was a significant moderating effect of exposure duration at 24 h where for every 1-min increase in duration, the effect size decreased by 0.15 (95% CI − 0.06 to − 0.24; *p* = 0.001; Fig. 4b).

#### The Effects of CWI on Recovery of Flexibility Performance

CWI was not effective in the recovery of flexibility performance at 48 h following eccentric exercise (Table 2; small effect; *p* = 0.48, GRADE = moderate). There were no other timepoints able to be analysed.

CWI was not effective in promoting the recovery of flexibility performance following high-intensity exercise. Findings at 1 h, 24 h and 48 h were all non-significant (Table 2; *p* > 0.05; GRADE = low).

Due to low study numbers, regression analysis was unable to be completed for flexibility outcomes from any exercise modality at any timepoint.

#### The Effects of CWI on Recovery from Delayed Onset Muscle Soreness (DOMS)

**Fig. 5:**
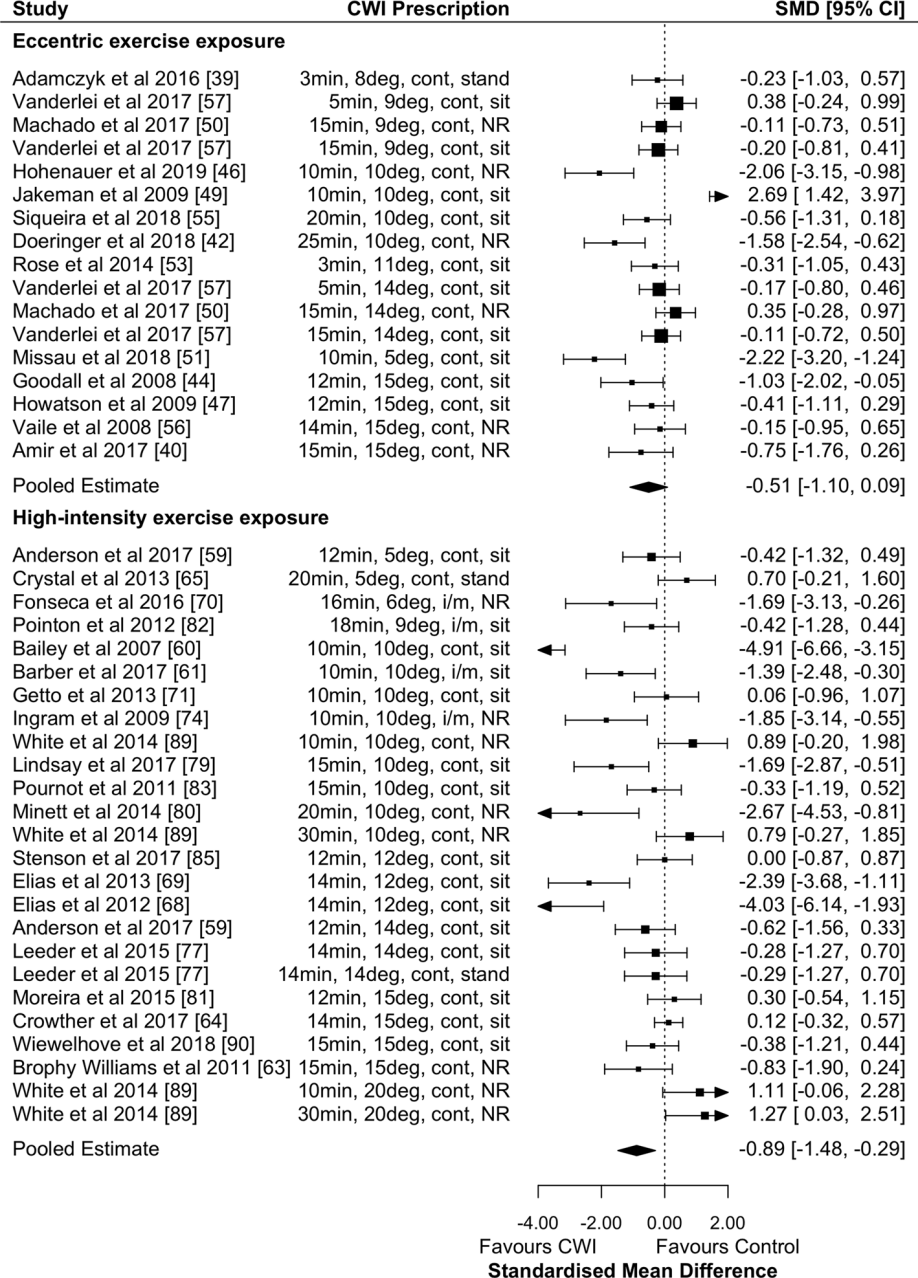
Forest plot illustrating the influence of CWI compared with passive recovery 24 h after exercise on muscle soreness (stratified by exercise intervention modality). *CI* confidence interval, *CWI* cold-water immersion, *SMD* standardised mean difference

CWI was not effective in reducing DOMS 24 h after eccentric exercise (Fig. 5; Table 2; small effect; *p* = 0.09; GRADE = moderate). CWI was effective at reducing DOMS after eccentric exercise at 48, 72, and 96 h (Table 2; all small effects; GRADE = high). Water temperature and exposure duration did not have significant moderating effects at any timepoint.

CWI was effective in reducing DOMS 24 h after high-intensity exercise (Fig. 5; Table 2; moderate effect; *p* = 0.003; GRADE = moderate). CWI had a moderate effect on reducing DOMS at 1 h and 48 h (Table 2; GRADE = moderate). Water temperature and exposure duration did not have significant moderating effects at any timepoint. There was a non-significant trivial effect in favour of passive recovery at 72 h, but insufficient studies to be meta-analysed at 96 and 168 h (Table 2).

#### The Effects of CWI on Perceived Recovery

CWI was not effective in increasing feelings of perceived recovery following eccentric exercise at 24 and 48 h (Table 2; trivial effect; GRADE = high) but did have small beneficial effects at 72 and 96 h (Table 2; GRADE = high). Water temperature or exposure duration did not have significant moderating effects at any timepoint.

CWI was effective in increasing feelings of perceived recovery 24 h following high-intensity exercise (Table 2; moderate effect; *p* = 0.001; GRADE = moderate). At 48 h, there was a non-significant trivial effect in favour of passive recovery compared with CWI (Table 2; GRADE = high). There were no significant moderating effects of water temperature or exposure duration at any timepoint.

#### The Effects of CWI on Recovery of Creatine Kinase

**Fig. 6:**
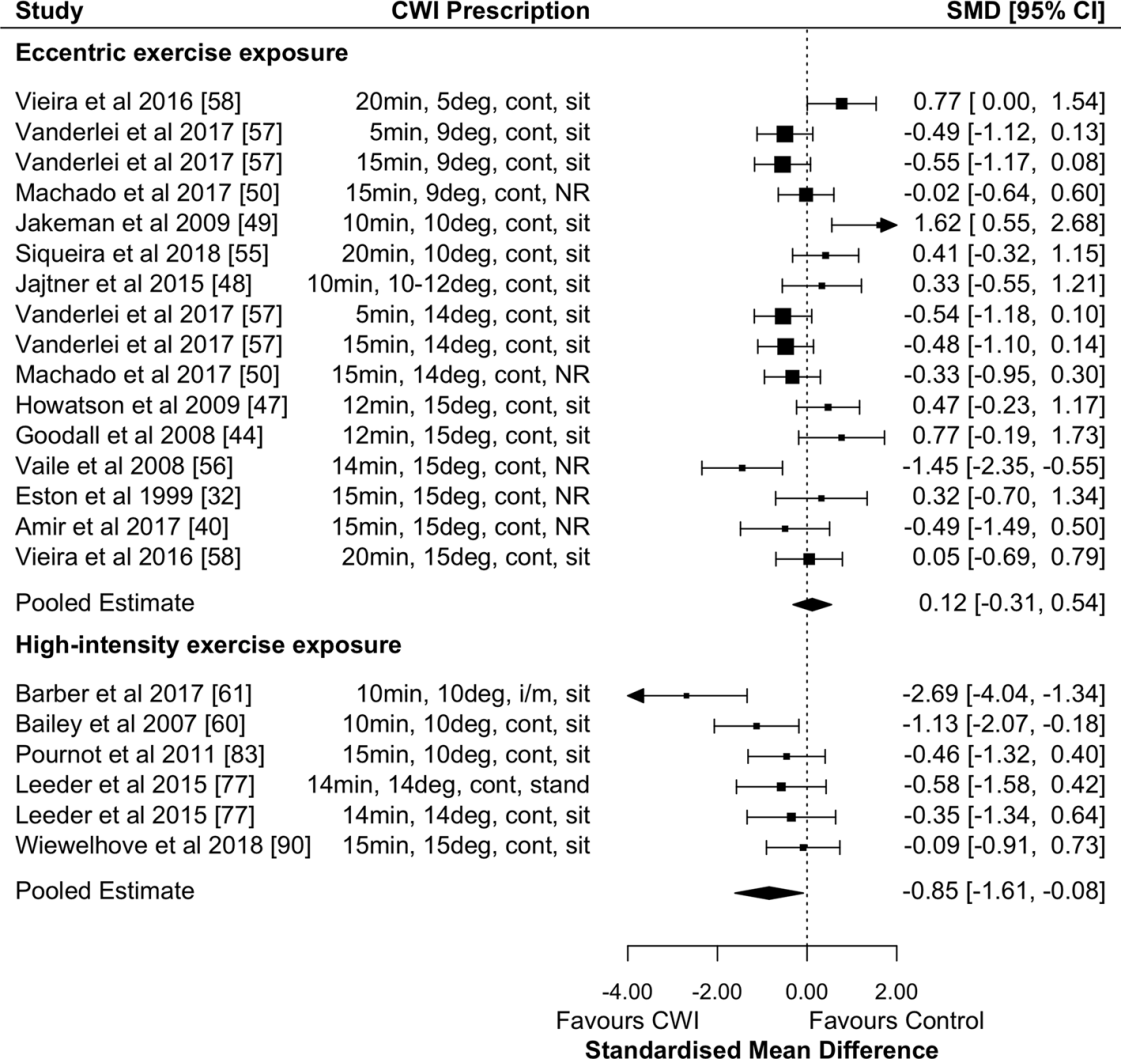
Forest plot illustrating the influence of CWI compared with passive recovery 24 h after exercise on removal of CK (stratified by exercise intervention modality). *CI* confidence interval, *CK* creatine kinase, *CWI* cold-water immersion, *SMD* standardised mean difference

CWI was not effective in reducing circulating CK 24 h after eccentric exercise (Fig. 6; trivial effect; *p* = 0.60; GRADE = moderate). The only significant timepoint where CK was reduced after eccentric exercise using CWI was at 168 h (i.e. 7 days) (Table 2; GRADE = moderate). Other timepoints showed non-significant trivial effects in favour of CWI (Table 2; GRADE 48 h and 72 h = moderate; GRADE 96 h = high), except for 1 h which showed large non-significant effects in favour of passive recovery (Table 2; GRADE 1 h = low). There were no significant moderating effects of water temperature or exposure duration at any timepoint.

CWI was effective in reducing circulating CK 24 h after high-intensity exercise (Fig. 6; Table 2; moderate effect; *p* = 0.03; GRADE = moderate). There was a significant exposure duration moderating effect at 24 h whereby for every 1-min increase in exposure duration, the effect size decreased by 0.28 (95% CI − 0.09 to − 0.47; *p* = 0.004; Fig. 4a). The only other timepoint that showed significant results after high-intensity exercise was at 48 h (Table 2; GRADE = moderate). There were significant moderating effects at 48 h of both water temperature and exposure duration, whereby for every 1-min increase in duration (Fig. 4b) and every 1 ℃ increase in temperature (Fig. 4d), the effect size for both duration and temperature decreased by 0.51 (95% CI − 0.25 to − 0.77; *p* < 0.001). There were insufficient studies to conduct meta-analyses for the other timepoints.

## Discussion

The aim of the present review was to examine the efficacy of CWI for promoting the recovery of numerous physiological, perceptual and athletic performance variables, as well as attempt to identify dose–response relationships between CWI temperature and/or duration with outcome measures through meta-regression. Overall, results were mixed, but some key findings were evident. CWI was effective at positively influencing power performance for both eccentric and high-intensity exercise. CWI was also effective at reducing CK concentrations as well as reducing DOMS and increasing perceived recovery from both eccentric and high-intensity exercise. CWI dose–response relationships involving water temperature and/or exposure duration were evident after high-intensity exercise and indicated that shorter time and lower temperatures were related to the largest effects on serum CK concentrations (duration and temperature dose effects), and endurance performance (duration dose effects only).

To the authors’ knowledge, this is the first review to compare CWI and passive recovery and their effects on recovery of physiological, perceptual and athletic performance measures at specific timepoints following differing exercise interventions in athletic populations, making it it most relevant to the individuals that use this recovery method the most. Despite using a narrower search strategy than previous reviews, this review identified the greatest number of studies included in CWI meta-analyses. This is also the first review to use meta-regression to determine significant dose–response relationships between water temperature and/or exposure durations, and various outcome measures. Furthermore, this is also the first review to account for methodological variations within parallel and crossover study designs, which is important because this research area includes both parallel and crossover studies.

### Effect of CWI on Performance Measures

**Fig. 7:**
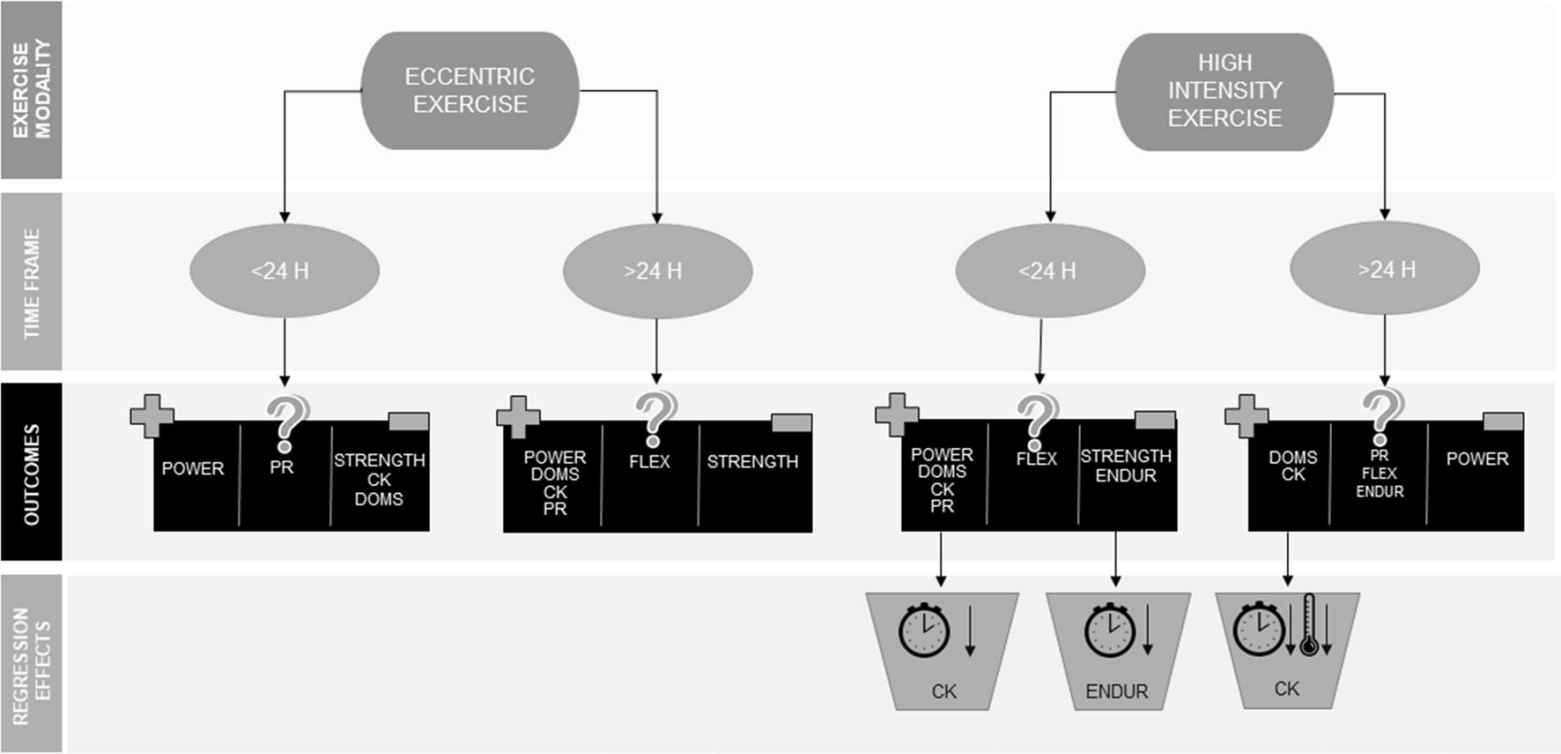
Summary of review outcomes presented to allow practitioners easy interpretation. *CK* creatine kinase, *DOMS* delayed onset muscle soreness, *Endur* endurance, *Flex* flexibility, *H* hours, *PR* perceived recovery

A variety of performance measures were utilised to allow practitioners to decide whether CWI is an appropriate method of recovery to influence performance (Fig. 7). These measures included muscular strength, flexibility, muscular power (such as sprint performance, jump performance and anaerobic power performance) and endurance performance. There was some variability in the influence of CWI on the outcome measures between eccentric and high-intensity exercise, potentially due to the different nature of exercise [8]. The variability of the influence of CWI on the outcome measures may also be accounted for by the training status of the participants; physically active participants may have less muscle damage induced through exercise than their untrained counterparts [91].

#### Following Eccentric Exercise

Pooled results from eccentric exercise studies show that muscular power performance but not strength was mostly likely to be significantly positively influenced using CWI after eccentric exercise. This allows some hypotheses to be drawn in terms of how CWI influences muscular physiology. There is the potential that CWI may specifically impact powerful dynamic movements rather than static strength [8]. Most studies (6/8) included in the recovery of muscular strength analysis used isometric strength testing [43, 46, 50, 55, 56, 58], and as isometric strength is slower to develop force than dynamic power activities because of the lack of a stretch–shortening cycle, so force production is more reliant on the stiffness of the musculo-tendinous system [92]. Research reports that increased musculo-tendinous stiffness increases isometric force, whereas reduced musculo-tendinous stiffness increases the performance of movements reliant on the stretch–shortening cycle [93]. Considering that dynamic power movements are reliant on the stretch–shortening cycle producing force during eccentric and concentric phases of movement, there is the possibility that CWI reduces musculo-tendinous stiffness within the body, explaining why dynamic muscular power is more positively influenced by CWI than isometric muscular strength. However, it should be noted that some strength outcomes had positive effect sizes but were non-significant due to study variability. The non-significant positive results for strength recovery contrasts with a previous review that concluded that cooling of the neuromuscular system inhibits isometric muscular strength [94]. In addition, cooling of the neuromuscular system reduces central nervous system fatigue [95], which could be why dynamic muscular performance has been significantly improved following CWI and strength results were non-significant (the difference could be the number of studies that used static strength (isometric measures) compared with dynamic strength (concentric or eccentric measures).

CWI did not influence flexibility; however, the limited number of studies (< 2) makes it difficult to draw definitive conclusions.

There were no eccentric studies that examined the effect of CWI on endurance performance.

#### Following High-Intensity Exercise

Pooled effects from high-intensity exercise interventions showed that muscular power but not muscular strength was significantly enhanced 24 h after exercise. This is partially consistent with data from previous eccentric exercise literature, showing that dynamic power activities may be most favourably influenced by CWI reducing musculo-tendinous stiffness [92, 93]; however, this was only evidenced at 24 h post-exercise. There were only three studies (two of which used isometric testing [65, 83]) investigating the recovery of muscular strength following high-intensity exercise that found a non-significant trivial effect in favour of passive recovery 1 h and 24 h after exercise. This is in line with the findings for post-eccentric exercise, which could further indicate the influence of CWI on musculo-tendinous stiffness.

The null results shown for the recovery of endurance performance 24 h after CWI may be the result of the differences in the exercise protocols performed prior to CWI and subsequent performance testing. The studies that performed steady state aerobic protocols prior to CWI found positive effects of CWI during their steady state aerobic performance tests (i.e. 5-km runs) [62, 85]. However, studies that performed more anaerobic-based exercise protocols (i.e. intermittent repeat sprint ability) prior to CWI and endurance performance testing did not find in favour of CWI [63, 76, 88]. It is possible that the vasoconstriction induced by CWI redirects blood flow to the core and increases central blood volume [96]. This increase in central blood volume would lead to increases in stroke volume and cardiac output [97], which would increase aerobic performance. However, the null results for studies using anaerobic-based, repeat-effort exercise protocols prior to CWI could indicate that the glycolytic metabolic by-products increased acidity (and therefore muscle damage) in the muscle [98] to levels greater than what could be effectively cleared using hydrostatic pressure and vasoconstriction seen during CWI [95], resulting in decreased performance, regardless of the positive effects on cardiac dynamics. Further research is required to determine the potential of this hypothesis.

Results from the meta-regression show that shorter duration CWI (~ 12 min) may positively influence endurance performance more than longer durations (14–15 min) 24 h after exercise. It is also possible that the temperature of the immersions influenced the null results seen, as lower immersion temperatures result in decreased muscle temperature which leads to decreased blood flow, swelling and oedema [99]; all but one study used a temperature of 15 ℃, and meta-regression was therefore not possible due to the lack of variation. The very low GRADE rating for this timepoint indicates the high level of variability amongst the studies.

The limited number of studies assessing flexibility (< 3) makes it difficult to draw definitive conclusions from the data. The low GRADE rating evidences the variability within the data at all timepoints.

### Effect of CWI on Perceptual Measures

Delayed onset muscle soreness (DOMS) can range from muscle tenderness to severe debilitating pain that can impact subsequent athletic performance until the symptoms ease [100]. Perceived recovery indicates an athlete’s impression of how ready they feel for the next activity bout, with adequate recovery opportunities needed to balance the stress state to maintain or increase performance capacity [101]. There is the potential that perceptual measures could be influenced by an athlete’s belief in the treatment’s efficacy, which may alter their pain perception post-stimulus and provide a placebo effect [102].

#### Following Eccentric Exercise

Pooled results from eccentric exercise showed that CWI did not have significant beneficial effects on DOMS and perceived recovery until 48 h after exercise. The delayed onset of peak muscle soreness could explain why CWI did not show significant effects on recovery until after 48 h as athletes may not have experienced significant soreness until this point [103]. Feelings of perceived recovery peaked at the same timepoints, indicating a relationship between diminished feelings of muscle soreness due to CWI and greater feelings of recovery. It is not possible to discount possible placebo effects influencing study results as it is not possible to blind participants to the treatment [104].

#### Following High-Intensity Exercise

Pooled results from high-intensity exercise interventions demonstrated that CWI had an immediate effect on reducing DOMS and increasing feelings of recovery with significant moderate to large effects evident between 1 and 48 h after exercise. High-intensity exercise has been found to induce high levels of muscle soreness regardless of training status [105], and the decrease in inflammation and oedema induced by the hydrostatic pressure of CWI combined with the analgesic effects of cooling have been shown to reduce DOMS and increase perceived recovery [6, 95]. However, as seen in the eccentric exercise studies, it is not possible to discount possible placebo effects influencing study results as it is impossible to blind participants to the treatment [104].

### Effect of CWI on Creatine Kinase

Creatine kinase is a commonly used blood-borne biomarker that indirectly implies muscle damage following strenuous exercise [106]. Serum CK measures represent relative amounts of CK released, the degree of enzyme activity of the released CK and the rate of clearance from the serum [107]. The variation between increases in CK following exercise suggests that the appearance of CK may not be entirely representative of muscle cell damage [108] and could be impacted by sex, ethnicity and age [109].

#### Following Eccentric Exercise

Pooled results from eccentric exercise protocols showed that CWI did not influence the removal of CK from the blood at any timepoint, except for 168 h after exercise. Research has reported that the greatest rise in CK concentration occurs following moderate to high intensity eccentric exercise with multiple exercises and multiple sets, which encourages athletes to exercise to contractile failure [110, 111]. The studies included in the analysis rarely followed protocols where athletes exercised to failure and only used a single exercise rather than following a protocol that used multiple modalities of exercise that target the same muscle groups (i.e. incorporating push and pull exercises into ‘supersets’). It is therefore possible that the single exercise protocols used may not have been sufficiently intense to produce significant rises in CK when comparing CWI with passive recovery. This follows a trend identified by Callegari et al. [110], who reported that less intense eccentric exercise resulted in smaller increases in CK levels in the blood 24 h after exercise following passive recovery. There is also the possibility that the muscle group subjected to exercise is a factor in the CK response to exercise; research reports that upper body eccentric exercise produces more CK than lower body eccentric exercise [112], possibly due to the training status of the participant and the repeat bout effect where participants are used to performing lower body eccentric movements (i.e. jumping, squatting) in everyday life and physical training [113]. The lone significant result found at 168 h after exercise shows that CWI has the potential to reduce CK; the two studies [55, 58] (with three observation points) found that significantly reducing CK levels may take 7 days following eccentric exercise.

#### Following High-Intensity Exercise

Pooled results from high-intensity exercise interventions showed that using CWI resulted in significant lower CK concentrations at 24 and 48 h after exercise. The studies included in this analysis used similar protocols that included high-intensity running as well as team sport simulations that place high levels of stress on the lower limbs. It should be noted that high-intensity exercise is more representative of athletic training when compared with resistance-based exercise. High-intensity running-based exercise results in the greatest rise in CK levels after exercise when compared with lower intensity running [110]; the eccentric nature of running combined with high volumes of running may increase muscle damage (and therefore CK levels) in the lower limbs due to the stretch–shortening cycle that occurs within the muscles [114]. The dose–response relationship for water temperature and immersion duration at 48 h where a lower temperature may be more effective at shorter durations may be explained by the colder temperatures reducing the efflux of CK from the muscles to the extracellular space via reduced vessel wall permeability [43] as well as reduced inflammation resulting in less secondary tissue damage [4].

### Limitations and Future Research

This review has limitations that should be considered when interpreting the findings. The majority of studies used only isometric testing as a measure of strength, so it is not clear if other measures of strength (i.e. one-repetition maximum concentric or eccentric contractions) may be more positively influenced by CWI. Very few studies investigated the effects of CWI on endurance and flexibility performance, and therefore conclusions based on these variables should be interpreted with caution. This review identified only four studies that used female-only cohorts, compared with 44 studies that used male-only cohorts and six studies that used a mixed cohort. As a result, findings from this review might be more reflective of male rather than female responses to CWI.

It is important to highlight the limitations identified through risk of bias. Blinding of participants and researchers is not possible due to the nature of the treatments, which could enhance the potential of the placebo effect of CWI. The limited randomisation of treatment groups could also influence the results, especially in parallel studies where participants are only undertaking one treatment. Incorporating a CWI placebo in addition to the treatment groups may limit the influence of the placebo effect.

Further research should be conducted using female-only cohorts to address the current sex bias in CWI literature; this would address differences in physiology between males and females and enable the identification of appropriate recovery protocols for female athletes. In addition, future research could elucidate whether inter-individual differences (i.e. body morphology, fat and lean body mass) influence the effectiveness of CWI as a recovery protocol. Also, studies investigating the effects of CWI on the recovery of muscular strength should consider various measures of strength rather than isometric testing only to better discern the efficacy of CWI for recovering muscular strength. Given the positive relationships identified in this review, more studies identifying the effects of CWI after high-intensity exercise are needed to further evaluate the effects on endurance performance and flexibility. In addition, evaluations assessing whether participants believing that CWI will aid their recovery influences outcomes should be performed to identify potential belief and placebo effects.

## Conclusions

The present systematic review and meta-analyses identified 52 randomised controlled studies investigating the effect of CWI on the recovery of physiological, perceptual and performance outcomes. The findings indicate several benefits of using CWI as a recovery intervention, particularly following high-intensity exercise. A reduction in DOMS and improvements in perceived recovery for both eccentric and high-intensity exercise were observed after CWI. CWI is more likely to positively influence dynamic power movements rather than static strength following both eccentric and high-intensity exercise. Dose–response relationships emerged for several variables indicating that lower durations and temperatures may improve the efficacy of CWI if used after high-intensity exercise.

## Supplementary Information

Below is the link to the electronic supplementary material.

Supplementary file1 (PDF 235 kb)

Supplementary file2 (XLSX 18 kb)

Supplementary file3 (XLSX 15 kb)

Supplementary file4 (XLSX 10 kb)

Supplementary file5 (XLSX 11 kb)

Supplementary file6 (XLSX 19 kb)

Supplementary file7 (XLSX 13 kb)

Supplementary file8 (XLSX 17 kb)

## Data Availability

The datasets generated during and/or analysed during the current systematic review are available in the Online Supplements 2–8, see ESM.
