# Supplementary material for: Impact of Cold-Water Immersion Compared with Passive Recovery Following a Single Bout of Strenuous Exercise on Athletic Performance in Physically Active Participants: A Systematic Review with Meta-analysis and Meta-regression
Source: Sports Med. 2022 Feb 14;52(7):1667–88. doi: 10.1007/s40279-022-01644-9 (PMC9213381; doi:10.1007/s40279-022-01644-9)
Supplement: Supplementary file 1 — Supplementary file1 (PDF 235 kb) [file 40279_2022_1644_MOESM1_ESM.pdf]

Impact of cold-water immersion compared to passive recovery following a single bout of strenuous exercise on athletic performance in trained participants: A systematic review with meta-analysis and meta-regression; Sports Medicine; Emma Moore (University of South Australia), Joel T Fuller (Macquarie University), Jonathan D Buckley (University of South Australia), Sienna Saunders (University of South Australia), Shona L Halson (McAuley at Banyo), James R Broatch (Victoria University), Clint R Bellenger (University of South Australia); Corresponding Author: emma.moore@mymail.unisa.edu.au

Risk of Bias

Green = Yes; Yellow = Can't Say; Red = No; Blue = Does Not Apply

#	Author, Year	Title	1.1 focused question	1.2 randomised groups	1.3 concealment	1.4 blinded allocation	1.5 similar groups	1.6 treatment	1.7 outcomes	1.8 drop out	1.9 analysis	1.10 comparable results	2.1 Final Ranking
1	Adamczyk et al 2016	The Use of Thermal Imaging to assess the Effectiveness of Ice Massage and CWI as methods for supporting post Exercise Recovery								0			+
2	Amir et al 2017	The effect of single bout of 15mins of 15C CWI on DOMS indictators								0			+
3	Anderson et al 2017	Effect of Cold (14C) V Ice (5C) water immersion on recovery from intermittent running exercise								0			+
4	Argus et al 2017	CWI and CWT: No improvement of short-term recovery after resistance training								0			+
5	Bailey et al 2007	Influence of CWI on indices of muscle damage following prolonged intermittent shuttle running								0			++
6	Barber et al 2017	The efficacy of repeated CWI on recovery following a simulated rugby union protocol								0			+
7	Bosak et al 2009	Impact of CWI on 5km racing performance								0			-
8	Brophy-Williams et al 2011	Effect of immediate and delayed cold water immersion after a high intensity exercise session on subsequent run performance								0			-

#	Author, Year	Title	1.1 focused question	1.2 randomised groups	1.3 concealment	1.4 blinded allocation	1.5 similar groups	1.6 treatment	1.7 outcomes	1.8 drop out	1.9 analysis	1.10 comparable results	2.1 Final Ranking
9	Crowther et al 2017	Influence of recovery strategies upon performance and perceptions following fatiguing exercise: a RCT								5			+
10	Crystal et al 2013	Effect of cryotherapy on muscle recovery and inflammation following a bout of damaging exercise								0			+
11	Dantas et al 2019	CWI does not accelerate performance recovery after 10km street run: RCCT								0			++
12	Delextrat et al 2013	Effects of sports massage and intermittent CWI on recovery from matches by basketball players								0			+
13	Doeringer et al 2018	The effects of postexercise cooling on muscle performance and soreness perception								0			++
14	Elias et al 2012	Effects of water immersion on post training recovery in Australian footballers								0			+
15	Elias et al 2013	Effectiveness of water immersion on postmatch recovery in elite professional footballers								0			+
16	Eston et al 1999	Effects of CWI on the symptoms of exercise induced muscle damage								0			+
17	Fonseca et al 2016	Use of CWI to reduce muscle damage and DOMS and preserve muscle power in Jiu-Jitsu athletes								4			+
18	Getto et al 2013	Comparison of active recovery in water and CWI after exhaustive exercise								7			+
19	Goodall et al 2008	The effects of multiple CWI on indices of muscle damage								0			+
20	Hassan 2011	Thermal therapy and DOMS								0			+
21	Higgins et al 2013 (1)	Acute response to hydrotherapy after a simulated game of rugby								0			+

#	Author, Year	Title	1.1 focused question	1.2 randomised groups	1.3 concealment	1.4 blinded allocation	1.5 similar groups	1.6 treatment	1.7 outcomes	1.8 drop out	1.9 analysis	1.10 comparable results	2.1 Final Ranking
22	Higgins et al 2013 (2)	Evaluation of hydrotherapy, using passive tests and power tests, for recovery across a cyclic week of competitive rugby union								0			+
23	Hohenauer et al 2019	Partial body cryotherapy (-135) and CWI (10) after muscle damage in females								2			+
24	Howatson et al 2009	The influence of CWI on adaptation following a single bout of damaging exercises								0			+
25	Ingram et al 2009	Effect of water immersion methods on post-exercise recovery from simulated team sport exercise								0			+
26	Jajtner et al 2015	Comparison of the effects of electrical stimulation and CWI on muscle soreness after resistance exercises								0			+
27	Jakeman et al 2009	A single 10min bout of CWI therapy after strenuous plyometric exercise has no beneficial effect on recovery from the symptoms of exercise-induced muscle damage								0			+
28	Jones et al 2013	The effects of different recovery interventions following a repeated rugby union (sevens) game simulated protocol								2			+
29	Lane et al 2004	Effect of selected recovery conditions on performance of repeated bouts of intermittent cycling separated by 24 hours								0			+
30	Leeder et al 2015	Effects of seated and standing CWI on recovery from repeated sprinting								0			++
31	Leeder et al 2019	CWI improves recovery of sprint speed following a simulated tournament								0			+

#	Author, Year	Title	1.1 focused question	1.2 randomised groups	1.3 concealment	1.4 blinded allocation	1.5 similar groups	1.6 treatment	1.7 outcomes	1.8 drop out	1.9 analysis	1.10 comparable results	2.1 Final Ranking
32	Lindsay et al 2017	The physiological response to CWI following mixed martial arts training session								0			++
33	Machado et al 2017	Dosages of CWI post exercise on functional and clinical responses								0			++
34	Minett et al 2014	CWI decreases cerebral oxygenation but improves recovery after intermittent sprint exercise in the heat								0			+
35	Missau et al 2018	CWI and inflammatory response after resistance exercises								2			++
36	Moriera et al 2015	CWI did not accelerate recovery after a futsal match								0			+
37	Paddon-Jones et al 1997	Effect of cryotherapy on muscle soreness and strength following eccentric exercise								0			+
38	Pointon et al 2012	CWI recovery after simulated collision sport exercise								0			+
39	Pournot et al 2011	Short term effects of various water immersions on recovery from exhaustive intermittent exercise								0			+
40	Rose et al 2014	Does WBC improve muscle recovery after damaging eccentric exercise?								0			+
41	Rupp et al 2012	The effect of CWI on 48h performance testing in collegiate soccer players								0			+
42	Sanchez-Urena et al 2018	Effectiveness of 2 CWI protocols on neuromuscular function recovery: a tensiomyography study								0			++
43	Siqueira et al 2018	Multiple CWI attenuate muscle damage but not alter systemic inflammation an muscle function recovery: a parallel RCT								1			+
44	Stenson et al 2017	5000m run performance is not enhanced 24hrs after an intense exercise bout and CWI								0			-

#	Author, Year	Title	1.1 focused question	1.2 randomised groups	1.3 concealment	1.4 blinded allocation	1.5 similar groups	1.6 treatment	1.7 outcomes	1.8 drop out	1.9 analysis	1.10 comparable results	2.1 Final Ranking
45	Tabben et al 2018	CWI enhanced athletes wellness and 10m short sprint performance 24h after a simulated mixed martial arts combat								0			+
46	Takeda et al 2014	The effects of CWI after rugby training on muscle power and biochemical markers								0			+
47	Vaile et al 2008 (1)	Effect of hydrotherapy on recovery from fatigue								0			+
48	Vaile et al 2008 (2)	Effect of hydrotherapy on the signs and symptoms of DOMS								0			+
49	Vanderlei et al 2017	Post exercise recovery of biological, clinical and metabolic variables after different temperatures and durations of CWI: a RCT								6			++
50	Vieira et al 2016	The effect of water temperature during CWI on recovery from exercise induced muscle damage								0			++
51	White et al 2014	The effect of various CWI protocols on exercise induced inflammatory response and functional recovery from high intensity sprint exercise								0			++
52	Wiewelhove et al 2018	Effects of different recovery strategies following a half marathon on fatigue markers in recreational runners								0			-
